# Agent-Supported Peer Collaboration in MOOCs

**DOI:** 10.3389/frai.2021.710856

**Published:** 2021-09-10

**Authors:** Stergios Tegos, Apostolos Mavridis, Stavros Demetriadis

**Affiliations:** Software and Interactive Technologies Lab, School of Informatics, Aristotle University of Thessaloniki, Thessaloniki, Greece

**Keywords:** MOOC, conversational agent, collaborative learning, support, interaction

## Abstract

While massive open online courses (MOOCs) can be effective in scaling education, orchestrating collaborative learning activities for large audiences remains a non-trivial task that introduces a series of practical challenges, such as the lack of adequate human support. Even when collaboration takes place, there is uncertainty whether meaningful interactions will occur among learners. This work presents the architecture of a prototype system called PeerTalk. The system was created to enable instructors to easily incorporate real-time collaborative learning activities into their online courses. Furthermore, PeerTalk employs a conversational agent service that aims to scaffold students’ online collaboration and provide valuable guidance, which can be configured by the course instructor. In order to investigate the user-acceptance of the system, two evaluation studies took place. The first one involved a group of experts, i.e., MOOC instructors who are expected to use such a system in their course, whereas the second study featured 44 postgraduate students. The study findings were encouraging in terms of the system efficiency and usability levels, laying the foundation for a conversational agent service, which can effectively scale the support of the teaching staff and be easily integrated in MOOC platforms, creating further opportunities for valuable social interaction among learners.

## Introduction

In the light of the pandemic, massive open online courses (MOOCs) have been established as one of the most important crisis management solutions, enriching learning opportunities and ensuring that education continues without major disruptions (Bylieva et al., 2020). Indeed, MOOCs have seen a surge in enrollments, refocusing attention on the opportunity to democratize higher education by providing open access to anyone in the world (Bhattacharya et al., 2020).

Video-based lectures still play a major role in presenting knowledge to MOOC students and it is often hypothesized that students’ initial knowledge base is constructed by attending video material (Ng and Widom, 2014). This knowledge is then reinforced by individually answering closed-type questions as part of quizzes or another type of automatically graded assignments such as coding exercises, leading to some form of corrective feedback. Although numerous MOOCs are still being viewed as informational landscapes, during the last few years, instructional designers have begun integrating opportunities for extensive social interactions in their MOOCs (Wang et al., 2018). This type of interaction is considered to positively impact the quality of the learning process at a cognitive and metacognitive level, boosting memory retention and assisting the development of metacognitive skills (Pifarre and Cobos, 2010). Moreover, social interaction can enhance students’ engagement levels at a motivational and affective level, increasing students’ interest and resulting in reduced drop-out rates. This is especially important since MOOCs inability to provide an interactive social environment with sustained support is often regarded as one of the main factors preventing MOOCs from reaching their highest potential (Demetriadis et al., 2018).

However, even when instructors decide to incorporate collaborative learning activities in their syllabus, there is no guarantee that meaningful interactions will occur among learners. A key postulate of computer-supported collaborative learning (CSCL) is that collaboration should be somehow scripted to increase the probability of constructive interactions taking place (Wang et al., 2017). Still, considering the limited teaching staff resources of most MOOCs, orchestrating and supporting collaborative activities are far from trivial tasks in real world settings, especially considering the practical issues that arise.

In an attempt to automate and scale human support, researchers have recently started to explore the usage of conversational agents to efficiently facilitate students’ collaboration in MOOCs (Tomar et al., 2017; Caballé and Conesa, 2018; Demetriadis et al., 2018). Conversational agents, often regarded as a subgroup of pedagogical agents, have a long history in the field of technology-enhanced learning, successfully serving a wide range of pedagogical roles, such as tutors, coaches or learning companions (Winkler and Söllner, 2018). The few studies conducted in this area have indicated that conversational agents can effectively support and enrich students’ collaboration (Caballé and Conesa, 2018). Such agents can be used to increase students’ engagement, minimize dropout rates, and amplify the peer support resources that are available (Ferschke et al., 2015).

Against the above, this study presents a prototype collaboration system, called PeerTalk. Drawing on a previous line of research on conversational agents (Tegos et al., 2019), PeerTalk integrates a configurable conversational agent service that aims to *1*) enable the easy integration of *ad-hoc* collaborative activities in MOOCs and *2*) provide automated facilitation and scaffold students’ collaborative learning. Furthermore, the system leverages the idea of “learning partners” in an attempt to overcome coordination issues, which are regarded as of the main practical challenges when students are asked to collaborate synchronously in the context of a MOOC (Tomar et al., 2017).

After introducing the PeerTalk system along with its architecture, the next sections present an exploratory study focusing on the usability and user-acceptance of the prototype system. The study features two phases: an expert-based evaluation, involving MOOC instructors, and a user-based evaluation, performed by actual learners who have used the system in the context of a small private online course.

## The PeerTalk Platform

PeerTalk was built to enable engaging co-browsing experiences among learning partners. Since the platform is intended to promote real-time collaboration in MOOCs and LMSs, maintaining high levels of interoperability was a top priority during its design and implementation.

**FIGURE 1:**
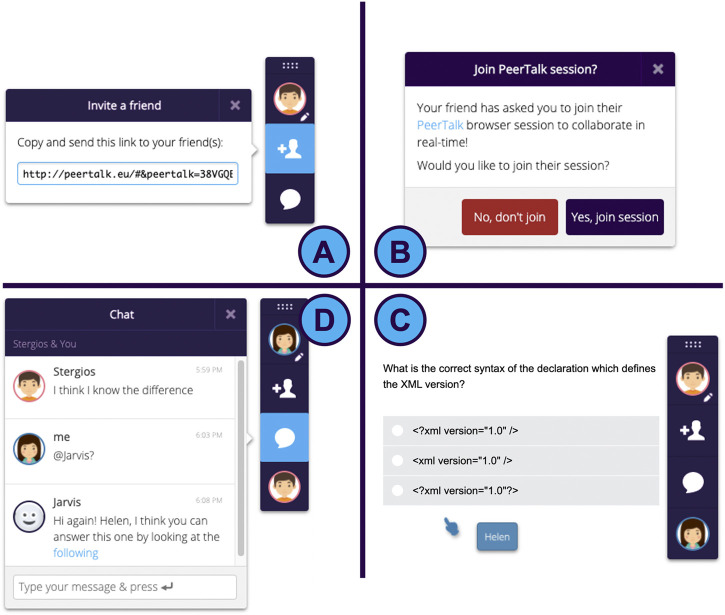
An illustration of the steps involved in starting a PeerTalk session and forming a team.

The collaboration among a group of learners begins instantly after initiating a PeerTalk session. More specifically, a learner can generate a link from the PeerTalk interface (Figure 1A) and send it to their learning partner. After the partner joins the PeerTalk session, they can collaborate in real-time since all course interactive elements are automatically synced among peers (Figure 1B), who can view each other’s cursor (Figure 1C), jointly interact with the interface of the learning environment and communicate *via* a chat (Figure 1D). A PeerTalk session may last over multiple web pages and, thus, it does not have to be interrupted when a user navigates on another course activity. While a PeerTalk session is active, learners can receive dynamic guidance by the PeerTalk conversational agent (Figure 1D).

The PeerTalk conversational agent was designed to be reusable in various courses and domains. In this manner, the agent domain model can be configured by any MOOC instructor, who may be interested in providing automated support to their students. The configuration of the conversational agent does not require any coding skills and can be accomplished by modifying Google Sheet templates. Those templates comprise a series of simple rules, consisting of a “trigger” and a “response.” The trigger is essentially an event, which may be a button click, a phrase discussed in the chat, or even the formation of a team, i.e. when a learner accepts an invitation to join a PeerTalk session. The agent response may include rich text, which appears simultaneously on the screens of both learning partners, when the respected trigger (event) is captured. Although the agent design is still a work-in-progress, the conversational agent currently leverages the Dialogflow natural language processing (NLP) engine in order to detect learners’ intents and display the relevant responses, set by the teacher/instructor.

Considering that human instructors cannot effectively scale their support to large audiences, such as the ones often found in MOOCs, the main goal of PeerTalk conversational agents is to provide valuable guidance and stimulate productive forms of dialogue, where peers discuss key domain concepts and build on each other’s contributions. For example, the conversational agent may intervene during peers’ discussion to display a tip or a challenging question, asking students’ opinion on an important course topic. When this occurs, peers may leverage their critical thinking and perform some mental rehearsal in order to try answering the agent question, thus reinforcing links in their mental models and enhancing memory retention.

The methodology employed for the development of the PeerTalk system was the ADDIE model, which is mostly used when creating educational systems and shares many similarities with the Agile software development model (Aldoobie, 2015). The first phase involved gathering input from educators and instructional designers about the necessary features of a synchronous collaboration tool. As a result, a semi-structured interview with various stakeholders was conducted to promote the compilation of the system functional and non-functional specifications. The next step was the design and development of the platform through the implementation of a series of individual modules. Overall, PeerTalk was designed to support the following key use cases: synced video lectures, collaborative assignments and quizzes, co-coding exercises, whiteboards, and other custom real-time applications.

**FIGURE 2:**
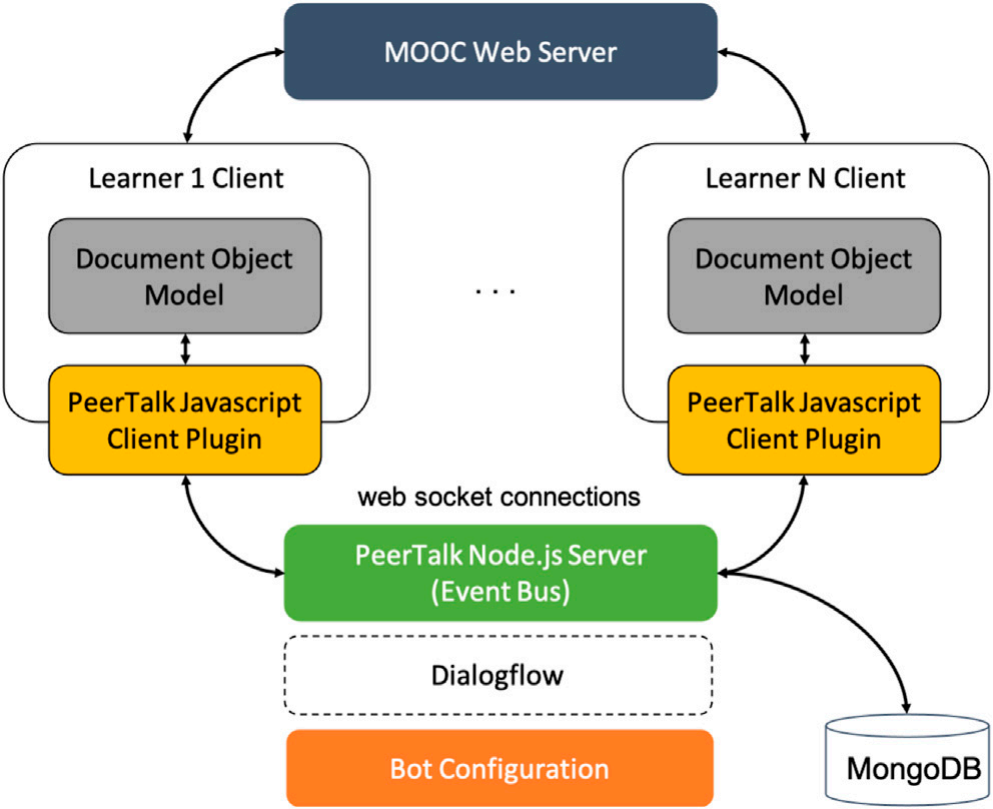
The architecture of the PeerTalk system.

The architecture that lies beneath the system comprises a server and a client module (Figure 2). The system is loosely coupled with the MOOC platform and adopts an Event-driven model, which operates over web socket connections between the clients and the server. The client-side is implemented through a javascript library, which was designed to be easily embedded into any web page as an external plugin. This library is responsible for reading and modifying the clients’ DOM through a set of custom event handlers, listening for events of multiple types, such as focus events, keyboard and mouse-related events, as well as mutation events.

The server side is implemented via a Node.js server, which operates as a central broker, serving the role of an event bus. A mongoDB is utilized to store session related information. This architecture is suitable for reactive applications that are dynamically distributed and scalable. The implementation of the platform employs a broker topology, in which a central broker collects and processes data, and all of the broker’s subscribers receive events asynchronously. This decision of incorporating the application logic into the central broker was taken with the intention to improve interoperability by reducing the need for complex specifications on the part of the subscribers’ hosts.

## Methods

A study was conducted to investigate the effectiveness of the PeerTalk platform to facilitate collaborative activities in MOOCs. The nature of the evaluation was twofold, focusing on gathering insights regarding the platform from both experts in the domain of MOOCs and university students.

### Participants

The expert-based evaluation phase of the study involved eight MOOC instructors (two females), participating voluntarily. The instructors were Greek native speakers and their age ranged from 33 to 48 yr (M = 40.83, SD = 4.95). The first phase was followed by a user-based evaluation conducted in the context of a small private online course (SPOC), which was called “Design and development of educational software.” 44 students (18 females) enrolled in the SPOC as a part of their post-graduate computer science program. Therefore, students’ participation was mandatory. The ages of the students ranged from 23 to 56 yr (M = 28.05, SD = 7.65) and all of them were Greek native speakers. The goal of the SPOC was to familiarize students with the processes involved while building educational software. The course adopted a flipped classroom model and supplemented classroom teaching.

### Procedure

In the first phase of the study, the instructors were asked to interact with the PeerTalk system in the context of a demo course assignment. The assignment involved the participatory writing of an essay, emphasizing the problems students typically encounter while attending a MOOC. Any text written by the team was automatically saved every few seconds. This activity aimed at evaluating the platform so as to be able to accommodate student activities in an online learning environment. Initially, there was a presentation of the tool and its capabilities and then the instructors were given a description of the activity and a list of tasks to complete. The total duration of the activity was 1 h and after its completion followed the interview with the instructors, which lasted another 1 h.

During the second phase of the study, the system was evaluated by learners. The process began with a 5-min presentation of the system. Students were then informed about a task they had to complete as a team, in the context of an online educational activity. More specifically, the task expected learners to debate and submit a joint answer to an open question, relating to the multimedia principles that were previously discussed in class. After the completion of the activity, which lasted 45 min, students were asked to fill in two short questionnaires.

### Instruments

A heuristic evaluation was conducted by a group of experts, who were assigned with the following six tasks: *1*) create an invitation link for your partner, *2*) send the invitation link to your partner and wait for them to join the session, *3*) follow the guidelines of the assignment that is shown on your screen and collaborate with your partner to complete it, *4*) open the chat and communicate with your partner, *5*) utilize the chat to get information about the requirements of the assignment (through the conversational agent), *6*) close your session and then try to connect again with your partner. These tasks derived from the analysis of a previous pilot study, which revealed a usage scenario in real educational settings. The participants formed groups of two and worked together for the completion of the tasks. During this session, two authors marked whether the completion of each task was successful. There was a total agreement across all marks, while a computer application was used to measure the completion time of each task. Following this process, a semi-structured interview took place with each expert. The content of the interview was based on Weinschenk and Barker classification of heuristics (Weinschenk and Barker, 2000). Following each interview, two authors jointly coded all contributions and any disagreement was resolved through discussion. Afterwards, a series of frequency scores were calculated based on the participants’ responses.

During the user-based evaluation phase, the standardized System Usability Scale (SUS) was used to measure the perceived usability of the system (Tullis and Stetson, 2004). The specific tool was selected since it has been shown to provide reliable results even when used on small sample sizes. The SUS questionnaire consisted of 10 items, each asking respondents to express their agreement on a 5-point scale (1-strongly disagree to 5-strongly agree). Apart from the SUS questionnaire items, another questionnaire was also used to explore how the students perceived the agent presence. The questionnaire consisted of three questions, which mainly focused on investigating the agent’s usefulness, the timing of the agent interventions, and whether the agent had any interruption effect. Similarly to the SUS questionnaire, these items employed a 5-point Likert scale.

## Results

### Experts

The interviews with the experts resulted in the identification of the following key themes: *1*) the interface informs users about the results of their actions and the interface’s status (87.5%), *2*) the interface does not overload the user’s cognitive and visual limits (100%), *3*) the interface provides a satisfying user experience (100%), *4*) the interface provides additional assistance as needed or requested (87.5%), *5*) the interface is consistent (100%), and *6*) the interface makes users’ actions recoverable (12.5%).

During the experts’ heuristic evaluation, the task time was also measured providing useful information regarding efficiency relating to PeerTalk. The particular metric was quantified, calculating the time-based efficiency along with the overall relative efficiency (Albert and Tullis, 2013; Mifsud, 2019). Both efficiency values relate to the time a user requires to complete a particular task in the system. The time-based efficiency indicates the number of tasks a user can complete per second, whereas the overall relative efficiency reflects the ratio of the time spent by the users who successfully completed the task in relation to the total time spent by all users. The equation used for the time-based efficiency is the following:

$$\mathrm{Time-based efficiency}=\frac{\sum_{j=1}^{R}\sum_{i=1}^{N}\frac{n_{\mathrm{ij}}}{t_{\mathrm{ij}}}}{\mathrm{NR}}$$

In the aforementioned equation, the following variables are used:

N = The total number of tasks.

R = The number of users

n_ij_ = The result of task i by user j; if the user successfully completes the task, then N_ij_ = 1, if not, then N_ij_ = 0

t_ij_ = The time spent by user j to complete task I; if the task is not successfully completed, then time is measured until the moment the user quits the task.

The result of the calculation was 0.056 tasks/s.

Correspondingly, the measurement of the overall relative efficiency was based on the next equation:

$$\mathrm{Overall relative efficiency}=\frac{\sum_{j=1}^{R}\sum_{i=1}^{N}n_{\mathrm{ij}}t_{\mathrm{ij}}}{\sum_{j=1}^{R}\sum_{i=1}^{N}t_{\mathrm{ij}}}\;\times 100\%$$

The variables in the equation are the same as the aforementioned ones above. The result of this calculation was 96.5%. Currently, there are no available baseline values in the literature to compare the results of these calculations. This is logical because the measurements are highly dependent on the type of tasks, which can greatly differ among studies. Nevertheless, the reported values provide an estimation of the platform efficiency in a real-world scenario and their actual usefulness comes in handy in future evaluations of the platform, enabling comparisons with different user groups or systems offering similar functionality.

### Students

In order to investigate the internal consistency of the SUS scale, a Cronbach’s alpha *1*) analysis was performed. As expected, the SUS standardized scale was found to have a high reliability for our dataset; *α* = 0.93, N = 10. The participants evaluated the overall usability of the system with a SUS score of M = 89.60 (SD = 14.56). This result falls well above the SUS acceptable baseline since a SUS score that exceeds 85 is regarded as “excellent” (Bangor et al., 2009). Table 1 presents the results of each questionnaire item.

**TABLE 1:** The results for each item of the SUS questionnaire.

Questionnaire item ^(1-disagree, 5-agree)^	M	SD
1. I think that I would like to use this system frequently	4.66	0.61
2. I found the system unnecessarily complex	1.32	0.67
3. I thought the system was easy to use	4.48	0.76
4. I think that I would need the support of a technical person to be able to use this system	1.20	0.51
5. I found the various functions in this system were well integrated	4.36	0.92
6. I thought there was too much inconsistency in this system	1.23	0.60
7. I would imagine that most people would learn to use this system very quickly	4.64	0.72
8. I found the system very cumbersome to use	1.25	0.49
9. I felt very confident using the system	4.48	0.82
10. I needed to learn a lot of things before I could get going with this system	1.52	0.88

The results of the mini questionnaire that measured how well the students perceived the presence of the conversational agent are displayed in Table 2. The scale was found to have a high level of internal consistency, as determined by a Cronbach’s alpha of 0.82. Overall, students had a positive perception of the conversational agent that assisted them during the collaborative activity (M = 4.30, SD = 1.19).

**TABLE 2:** The results of the questionnaire regarding how the students perceived the agent presence.

Questionnaire item ^(1-disagree, 5-agree)^	M	SD	Agree^(4-5)^	Neutral (%)^(3)^	Disagree (%)^(1-2)^
1 (%). I found the PeerTalk agent (Jarvis) to be helpful for completing the activity	4.47	0.74	88.64	6.82	2.27
2. The PeerTalk agent messages were well-targeted (appeared at the right time)	4.28	0.85	81.82	11.36	4.55
3. The PeerTalk agent messages disrupted my collaboration with my partner(s)	1.70	1.19	11.36	9.09	77.27

## Discussion

The present study investigated the potential of the PeerTalk system to facilitate MOOC collaborative activities and mainly focused on the aspects of efficiency and usability. The study participants were eight MOOC instructors that undertook the role of experts and 44 university students, who were involved in the user-based evaluation. The evidence gathered from the expert-based evaluation was promising in terms of both the time-based and overall relative efficiency of the system. Moreover, the themes that derived from the interview with the instructors indicated an overall positive acceptance of the system. The issues that emerged mainly concerned the recoverability from unwanted actions and the participants regarded the possibility of editing and removing chat messages as a valuable addition.

The next study phase involved a user-based evaluation, which focused on evaluating the usability of the system as well as the student-agent interactions during an educational activity. The results obtained from the SUS questionnaire were positive, revealing that the students felt rather confident while using the system and perceived it as an easy-to-use tool. Furthermore, the interaction between the students and the agent was found to be beneficial. According to the students’ feedback, the agent helped them to complete the activity and did not have a major interruption effect on their collaboration. At this point, it should be taken into account that the agent was found to be mostly active during the first minutes of the activity and did not intervene very frequently due to its interval-based approach, which was blocking the delivery of consecutive agent interventions.

Overall, the data obtained from both experts and students demonstrate that the PeerTalk platform can be effectively used to promote collaboration without the risk of hindering the learning process or significantly increasing teachers’ burden. However, the findings of this study have to be seen in the light of some limitations. First, it should be noted that the study had a limited sample size and adopted a one-shot case study design. A more robust future study could employ a control group to investigate whether such a system can be beneficial in terms of enhancing learning outcomes. Second, the study outcomes cannot be generalized without further investigation because of the participants’ computer literacy level and motivation. Considering the activity was carried out in the context of a computer science postgraduate program, i.e., in a controlled environment, participants may have adjusted their conversational behavior to pay additional attention to the agent.

Still, we consider the findings of this feasibility study to be encouraging towards implementing an agent-based service that enables the deployment of ad-hoc collaborative activities featuring automated facilitation. Our vision is to lay the foundation for configurable and reusable conversational agent activities that promote and scaffold learners’ collaboration in the context of MOOCs. In this direction, a next study will explore more aspects of the agent operation as well as how teachers can configure and leverage the PeerTalk conversational agent service.

## Data Availability

The raw data supporting the conclusions of this article will be made available by the authors, without undue reservation.
